# Decreased utilization of allocentric coordinates during reaching movement in individuals with autism spectrum disorder

**DOI:** 10.1371/journal.pone.0236768

**Published:** 2020-11-18

**Authors:** Yumi Umesawa, Takeshi Atsumi, Reiko Fukatsu, Masakazu Ide

**Affiliations:** 1 Department of Medical Physiology, Faculty of Medicine, Kyorin University, Tokyo, Japan; 2 Department of Rehabilitation for Brain Functions, Research Institute of National Rehabilitation Center for Persons with Disabilities, Saitama, Japan; Universitat de les Illes Balears, SPAIN

## Abstract

Despite numerous reports of abnormalities in limb motor controls in spatial orientation in individuals with autism spectrum disorder (ASD), the underlying mechanisms have not been elucidated. We studied the influence of allocentric coordinates on ongoing reaching movements, which has been reported to strongly affect the reaching movements of typically developing (TD) individuals. ASD and TD participants observed a target presented randomly on one of the four corners of a frame on a screen. After it disappeared, another frame was presented slightly shifted leftward/rightward. The participants touched the memorized position of the target relatively congruent with a reference frame (allocentric condition) or ignoring it (egocentric condition). Results suggested that TD individuals were apt to touch the positions in allocentric manner rather than egocentric manner, while ASDs did not show this prioritization. Our findings demonstrate that decreased utilization of visual landmarks in ongoing movement may underlie motor disabilities in autism.

## Introduction

Individuals with autism spectrum disorders (ASD) often show poorer fine motor skills such as handwriting, drawing and ball handling [1]. Another study pointed out that handwriting difficulties in autistic children might be resulting from impairments in visuo-motor transformation [2]. While visual information of target object is useful in acquiring internal model for adaptive motor controls in typically developing (TD) children [3], children with ASD tended to put greater reliance on proprioceptive feedbacks in motor learning [4, 5]. This character was found to relate to reduced synaptic volume especially in the cerebellum which is a representative neural basis of internal models of motor control [6]. From these findings, different optimizing strategies of motor control in internal model would be responsible for difficulty to learn adaptive movements.

However, it is more essential to immediately and abruptly approach some objects in our daily life, before acquiring strategies for adequate movements. In these immediate movements, not only recognition of the object itself but also recognition of it in relation to surrounding environment of the object, that is, allocentric coordinates, is useful for efficient goal-directed reaching [7, 8]. Egocentric coordinates are another set of clues for reaching by locating the target in relation to the body, head, retina and so on. Previous studies showed that the reaching for targets were more accurate and less variable when the target location provided a context for allocentric coordinates [9–13].

A previous study reported impaired utilization of allocentric coordinates in relation to reduced third-person perspective taking to perceive outside space in individuals with ASD [14]. In line with the impairment of social cognition in ASD, such visual perspective taking (VPT) has been frequently discussed [15]. A large body of evidence also reported difficulties in limb control related to visuo-motor transformation in individuals with ASD [16–18]. Despite an increased focus on motor deficits in ASD [19], the utilization of allocentric representation in visual motor skills is not yet understood.

Several studies have tried to explain the neural substrates of allocentric and egocentric representations in TD children. In one study, allocentric spatial representation was suggested to be processed in the ventral stream, projecting from the primary visual cortex (V1) to the inferior occipito-temporal cortex, while the dorsal stream, projecting from V1 to the posterior parietal cortex (PPC), is assumed to process egocentric representation [20]. A meta-analysis of 48 diffusion tensor imaging (DTI) studies found that the brain in patients with ASD tended to have fewer white matter tracts that pass through the temporal lobe [21], which are thought to be involved in allocentric spatial representation. These findings suggest that impairments in allocentric representation in ASD might be derived from weakened neural networks extending from V1 to the inferior occipito-temporal cortex.

The present study sought to examine the characteristics of utilizing allocentric coordination during reaching movements in individuals with ASD. It has been demonstrated that in healthy individuals, background coordinates (i.e., reference frame [RF]) were also essential during immediate saccades and reaching towards a target [22, 23]. Thus, we applied a task where subjects reached for a target presented on a touch screen using the upper limb (i.e., right or left hand) to quantify the amount of bias suffered by movements of the allocentric RF in reference to an experimental paradigm used in a previous study [23]. The purpose of this study was to clarify whether individuals with ASD can be affected by allocentric coordinates in immediate goal-directed ongoing reaching that does not require motor learning. We hypothesized that individuals with ASD demonstrate reduced influence from allocentric coordinates on reaching movements because of greater reliance on egocentric coordinates.

## Materials and methods

### Participants

We recruited 17 individuals with ASD and 17 TD individuals (Table 1). ASD participants were recruited from parent groups for individuals with developmental disorders and the Department of Child Psychiatry in the Hospital of National Rehabilitation Center for Persons with Disabilities. We confirmed that two participants had concurrent ADHD with ASD. There was no significant between-group difference in age and handedness, which was assessed using the laterality quotient score of the Edinburgh Handedness Inventory [24]. The participants also completed the Japanese version of the Autism spectrum quotient (AQ) scale [25, 26], in which higher scores indicate stronger autistic traits. AQ scores in ASD group were significantly higher than those of the TD group (two-tailed *t* test: *t* (32) = 4.02, *p* = 0.0003, Cohen’s *d* = 1.38, 95% CI = [5.55, 16.31]). The Intelligence Quotients (IQs) were assessed using the Wechsler Adult Intelligence Scale-Third Edition (WAIS-III). There were no significant between-group differences in Verbal IQ (VIQ), Performance IQ (PIQ) and Full-scall IQ (FIQ), as well as all subcategories (i.e., verbal comprehension [VC], working memory [WM], perceptual organization [PO], and processing speed [PS]). We assessed handedness using the Laterality Quotient (LQ) score of the Edinburgh Handedness Inventory [24], and confirmed no group differences. An LQ score ≤ 0 indicates that participants have left laterality of hand/foot use in daily activity and could be regarded as left-handed. The present study was approved by Ethics committee of the National Rehabilitation Center for Persons with Disabilities, and all participants gave informed written consent before the experiment.

**Table 1:** Information of participants.

	ASD	TD
	N = 17	N = 17
**Sex (Male: Female)**	14: 3	10: 7
**Age**	20.9 ± 3.7 SD	19.5 ± 2.0 SD
**LQ**	57.9 (-78–100)	71.4 (-50–100)
AQ**	30.8 (13–43)	19.9 (7–32)
**VIQ**	111.2 (86–140)	116.2 (94–136)
**PIQ**	105.2 (72–140)	109.6 (80–137)
**FIQ**	109.1 (87–135)	114.7 (93–131)
**VC**	110.8 (80–141)	115.6 (88–147)
**PO**	103.9 (68–132)	109.7 (79–137)
**WM**	109.0 (83–151)	109.2 (81–137)
**PS**	100.2 (75–133)	110.5 (69–140)

Scores in each cell and parentheses denote the averages and ranges in ASD and TD groups, respectively. Asterisks denote statistical significance

(**p < 0.01).

Abbreviations: LQ, laterality quotient; AQ, autism spectrum quotient; VIQ, verbal IQ; PIQ, performance IQ; FIQ, full-scale IQ; VC, verbal comprehension; WM, working memory; PO, perceptual organization; PS, processing speed.

### Apparatus and general task procedures

**Fig 1:**
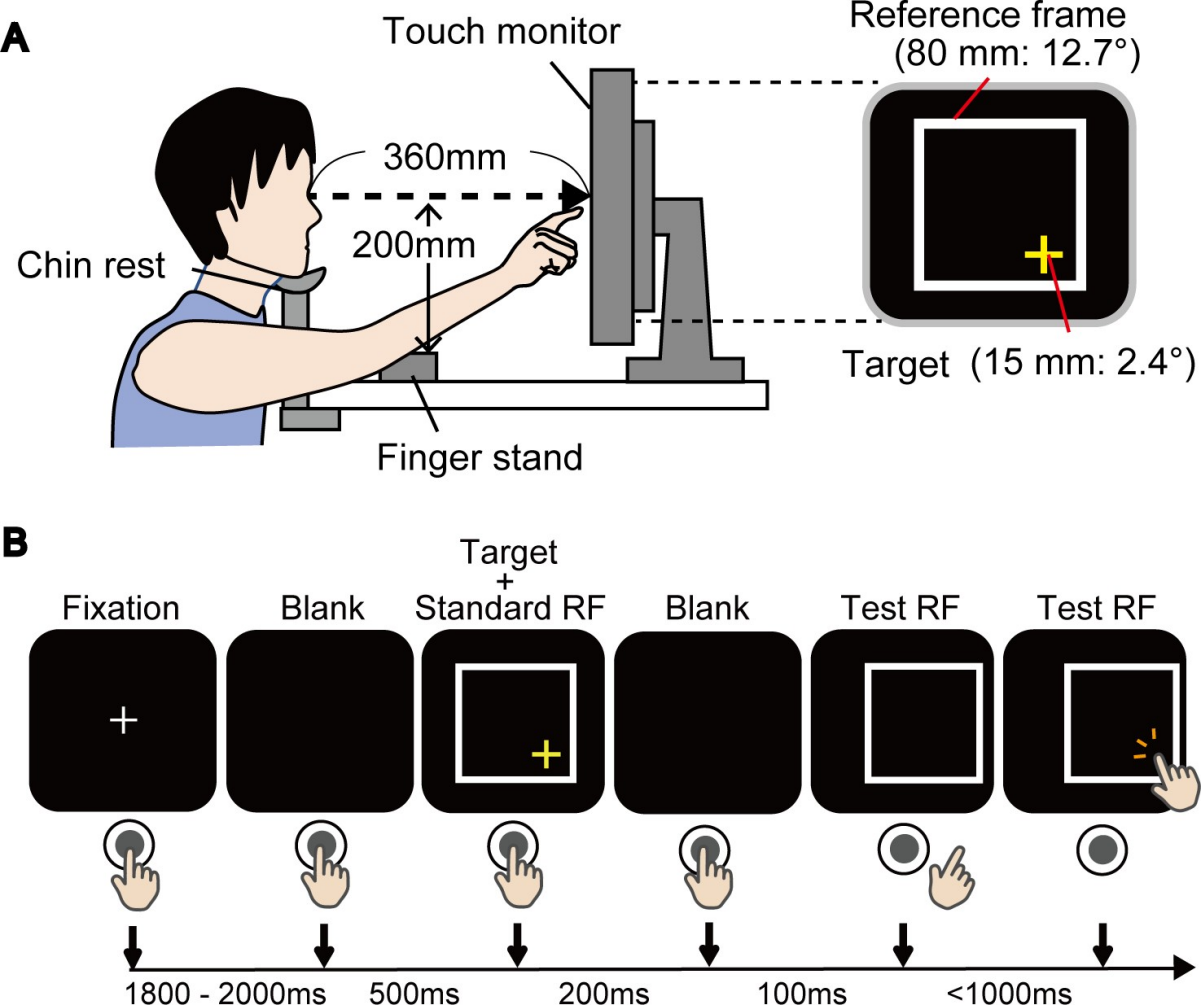
Experimental setup. **(A)** Experimental setups. Participants were seated facing a screen placed 360 mm in front of their eyes with their heads restrained by a chin rest. A yellow cross target was presented randomly on either of the four corners within a white square (i.e., reference frame: RF) on the screen. **(B)** Task procedures. Each column is the schematics of the visual field and the position of the hand relative to the button and the screen. A target with the Standard RF appeared for 200 ms proceeded by a blank of 500 ms and a fixation of random duration between 1800–2000 ms. After a blank (100 ms), only a Test RF shifted either leftward or rightward, or did not shift in Baseline trials. The participants touched a position on the screen according to instructions (see Materials and methods section) as quickly as possible (within 1000 ms). Test RF disappeared when the participants touched the screen.

According to the experimental paradigm in a previous study [23], the participants performed reaching movements for a target position in relation to a reference frame (RF) on a screen. The participants were seated facing a touch screen (ET1903LM-2UWA-1-WH-G, dimensions 376.32 mm (H) × 301.06 mm (V), Elo Touch Solutions, Inc.) with a resolution of 1280 pixels ×1024 pixels placed 360 mm in front of their eyes with their heads restrained by a chin rest (see Fig 1A). A customized PC (Predator G5900, Acer Inc.) and PsychoPy3 [27] were used to control the experiment. An index finger stand was positioned 200 mm below and 100 mm ahead of a subject’s eyes in the midsagittal plane. One ASD and two TD participants identified as being left-handed were instructed to use their left index fingers, while the others were instructed to use their right index fingers. A target (a yellow cross, 15 mm = 2.4° in visual angle) and a RF (Standard RF; a white square, 80 mm = 12.7°) appeared on a black background for 200 ms after a random delay (1800–2000 ms) followed by a blank (500 ms). The target cross was presented at a random location at one of the four corners of the frame. Another reference frame (Test RF) was displayed after a 500 ms blank in a position congruent with the Standard RF (Baseline trial) or shifted 20 mm (3.2°) either leftward or rightward in the horizontal plane with respect to the Standard RF. Baseline trials (20 times) were inserted in each of the *Egocentric* and *Allocentric conditions* to examine the possible influence of kinematic issues in ASD, especially regarding greater amounts of jerks in the reaching movement [28] performed in this task. The participants touched the position on the screen with their index finger soon after the Test RF was displayed according either to instructions: “Touch the disappeared target position ignoring Test RF” (*Egocentric condition*), or, “Touch the disappeared target position in Test RF relatively congruent with the position in Standard RF” (*Allocentric condition*). They were instructed to touch the target location as quickly and precisely as possible without any intentional corrections. They rested the finger on the finger stand within 500 ms after touch. It took 4–5 s to complete one trial (Fig 1B). Each of the *Egocentric* and *Allocentric conditions* included 60 trials, and it took 5–6 min to complete. The participants performed reaching tasks in both conditions on different days to avoid the influence of the effect of learning a strategy that is efficient for a specific condition (i.e., *Egocentric* or *Allocentric*). The orders of the conditions were counter-balanced among participants.

### Evaluation indexes

**Fig 2:**
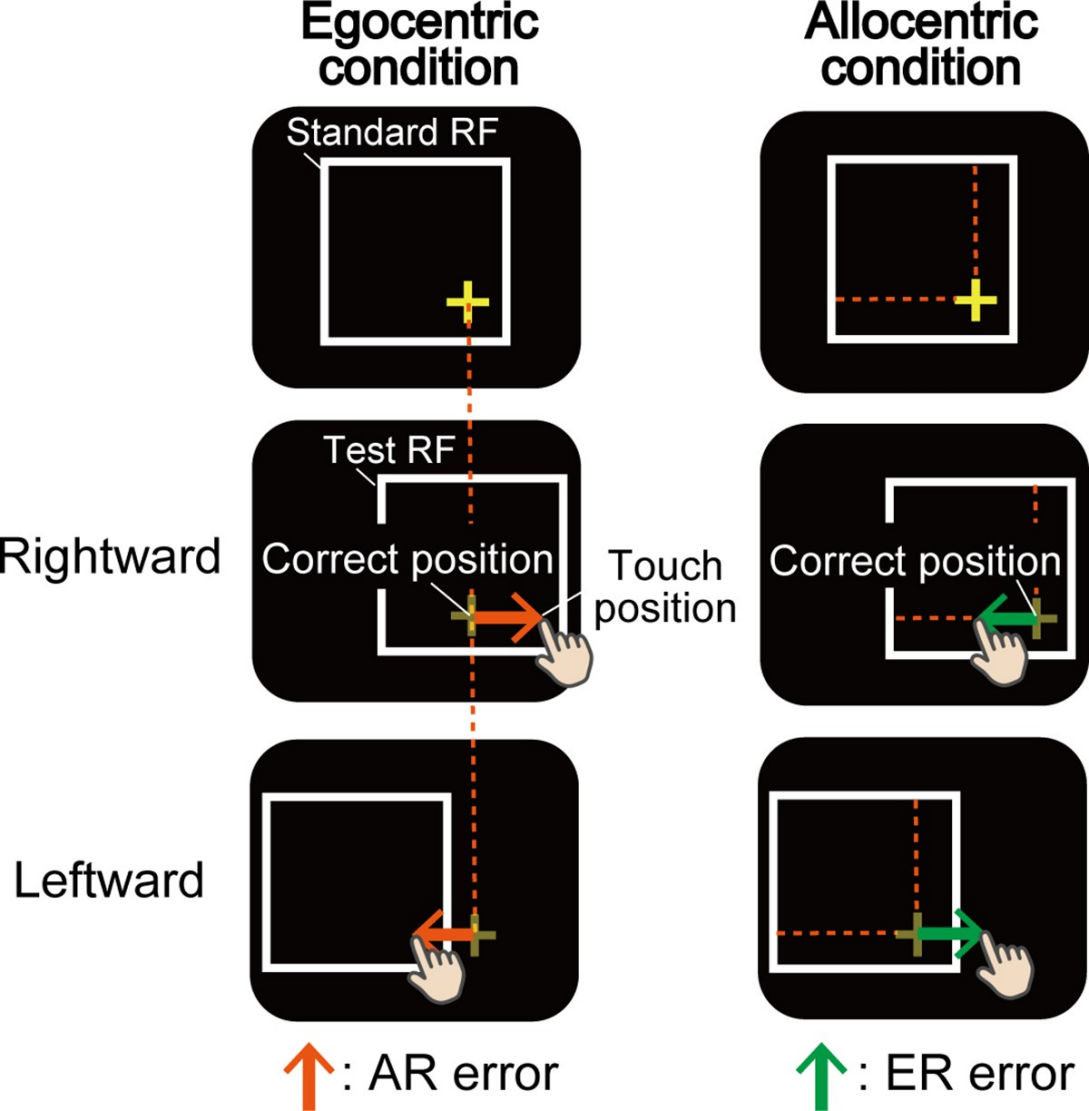
Evaluation indexes. We calculated the amount of error in X-axis to evaluate the influence of each coordinate’s conditions (i.e., *Egocentric* or *Allocentric conditions*) on the touch position. In the *Egocentric condition*, orange arrows indicate the amount of translation of the touch affected by allocentric representation (i.e., the target position in Standard RF) (AR errors). In the *Allocentric condition*, green arrows indicate the amount of translation of touch ‘*not*’ affected by allocentric representation, but rather affected by egocentric representation (ER errors).

Response times were calculated as time elapsed from the presentation of the Test RF to the timing of touch on the screen. We calculated the amount of errors in X-axis to evaluate influence of allocentric representation in the *Egocentric condition* (AR errors) and that of egocentric representation in the *Allocentric condition* (ER errors) on the touch position using following formula (see Fig 2).

1) 2) Rightward and Leftward errors in *Egocentric* (or *Allocentric*) *condition* were estimated as the degree how touch position in Test RF translated (or did not translate) toward memorized target position relatively congruent with Standard RF. 3) Total error (AR error / ER error) was the summation of both the errors. To confirm that the touch positions themselves were different between groups because of motor impairments that affect reaching movements, we calculated the size of X-axis errors in Baseline trials.

### Statistical methods

We used data that were obtained within 1 sec from the start of the measurement of responses for subsequent analysis. Student’s t-tests were used to compare response times in *Egocentric* and *Allocentric conditions* and the absolute error rate of touches in Baseline trials between groups. We performed one-sample *t* test versus zero in AR errors and ER errors to examine whether the translated touch position was affected by the allocentric or egocentric representations in the *Egocentric* or *Allocentric conditions*, respectively. A two-way ANOVA (2 × 2) was conducted to make between subjects (ASD or TD group) and within subjects (*Egocentric* or *Allocentric* coordinates condition) comparisons. The Smirnov-Grubbs test was performed to detect outliers with respect to touching errors. We calculated Cohen’s *d* and partial η^2^ to demonstrate effect size of the difference of the groups and the two-way ANOVA, respectively. We used SPSS Version 23.0 (IBM, New York, U.S.) to perform *t* test and ANOVA, and G*power 3.1 [29] to calculate the effect sizes.

## Results

**Fig 3:**
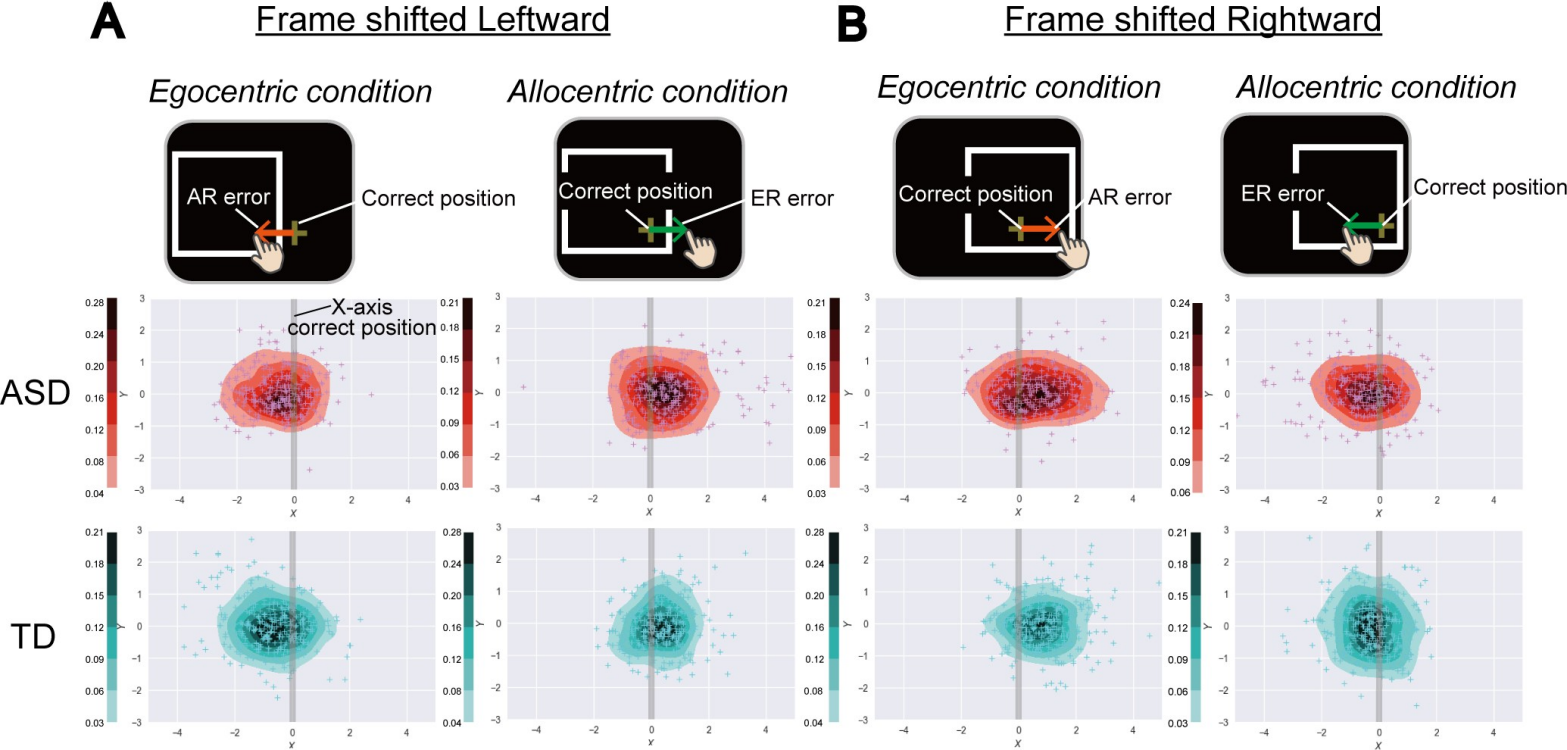
Touch positions on the screen when frame was shifted leftward or rightward. **(A)** Touch positions on the screen when frame was shifted leftward. X- and Y-axes indicates horizontal and vertical dimensions (deg) of the screen. Depth of colour (red: ASD, blue: TD) of the heat maps depicts the proportions of frequent touches in the area estimated by probability density function. Grey vertical lines indicate correct positions of touch according to instructions (allocentric or egocentric) in X-axis. **(B)** Touch positions on the screen when frame was shifted rightward. Others are same as above (A).

**Fig 4:**
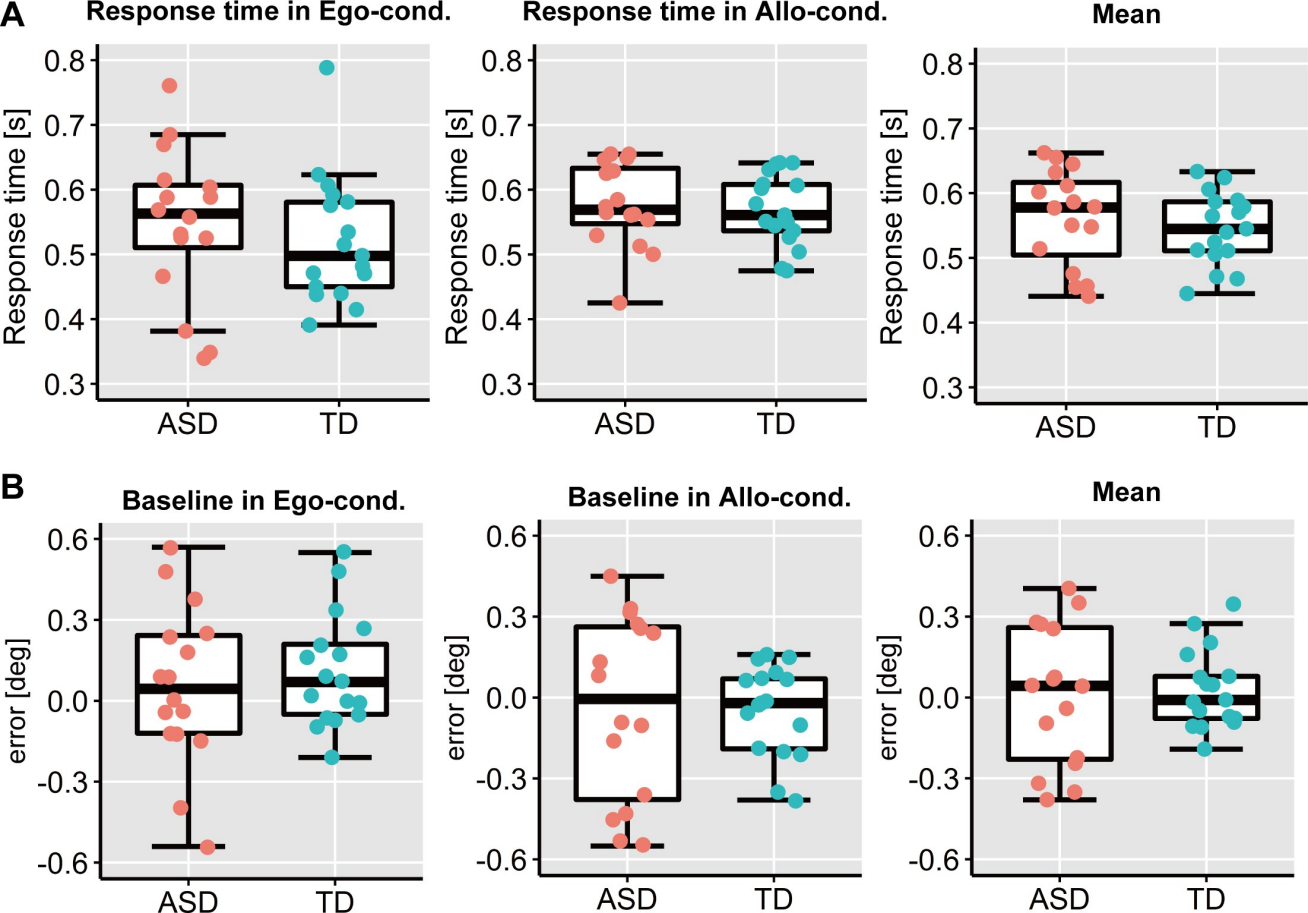
Response times and X-axis errors in baseline trials. **(A)** Response times in the *Egocentric condition*, *Allocentric condition*, and mean of the two conditions. The upper and lower boundaries of the standard boxplots represent the 25^th^ and 75^th^ percentiles. The horizontal line across the box marks the median of the distribution. The ends of vertical lines below and above the box represent the minimum and maximum, respectively. **(B)** X-axis errors in Baseline trials (i.e., no RF shift) in the *Egocentric condition*, *Allocentric condition*, and mean of those two conditions. Others are same as above (A).

Fig 3A and 3B depict touch positions in each condition. By calculating average of X-axis errors of touch position from correct position for each participant, we estimated AR errors (errors in the *Egocentric condition*) and ER errors (errors in the *Allocentric condition*). According to the Smirnov-Grubbs test, an outlier in average ER error was detected. Hence, we report the analysis results for all participants, excluding one ASD participant. There were no significant group differences in response times in either the *Egocentric* or *Allocentric condition* (*Egocentric* condition: *t* (31) = 0.67, *p* = 0.51, *d* = 0.23, 95% CI = [-0.05,1.02]; *Allocentric condition*: *t* (31) = -0.36, *p* = 0.73, *d* = 0.12, 95% CI = [-0.03, 0.05]) and mean response time of the two conditions (*t* (31) = 0.72, *p* = 0.48, *d* = 0.25, 95% CI = [-0.03, 0.06]; Fig 4A). Additionally, we did not observe a significant difference in absolute errors in Baseline trials between RF conditions *(Egocentric condition*: *t* (31) = -0.61, *p* = 0.55, *d* = 0.21, 95% CI = [-0.23, 0.13]; *Allocentric condition*: *t* (31) = 0.13, *p* = 0.90, *d* = 0.04, 95% CI = [-0.18, 0.20]) or mean absolute error in the Baseline trials of the two conditions (*t* (31) = -0.29, *p* = 0.77, *d* = 0.21, 95% CI = [-0.17, 0.13]; Fig 4B).

**Fig 5:**
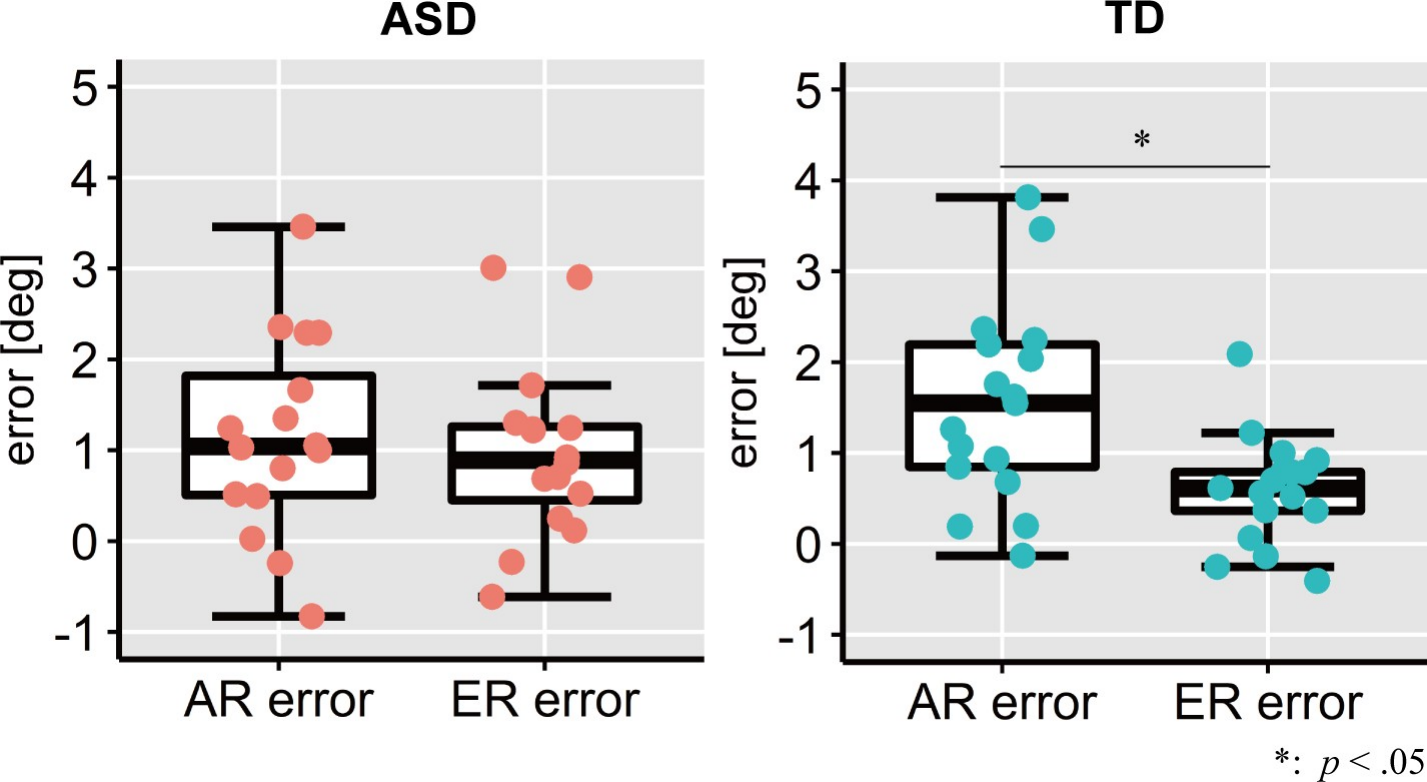
AR and ER errors in ASD and TD groups. The left box plot denotes AR errors and ER errors in the ASD group, and the right denotes those in the TD group. The upper and lower boundaries of the standard boxplots represent the 25^th^ and 75^th^ percentiles, respectively. The horizontal line across the box marks the median of the distribution. The ends of the vertical lines below and above the box represent the minimum and maximum, respectively. An asterisk denotes a significant difference between the two types of error (errors in *Allocentric* and *Egocentric conditions*).

Both AR and ER errors were significantly greater than zero in both ASD and TD groups (AR errors: *t*s (15, 16) = [4.24, 5.80], *p*s = [< 0.01, < 0.01], *d*s = [1.06, 1.41], 95% CIs = [{0.58, 1.74}, {0.97, 2.10}], in the ASD and TD groups, respectively; ER errors: *t*s (15, 16) = [4.05, 4.08], *p*s = [< 0.01, < 0.01], *d*s = [1.01, 0.99], 95% CIs = [{0.47, 1.51}, {0.28, 0.8}], in ASD and TD groups, respectively). Two-way repeated measures ANOVA showed a significant main effect of the coordinate conditions (*Allocentric* and *Egocentric*) (*F* (1, 31) = 8.41, *p* = 0.007, partial η^2^ = 0.21), but the main effect of group (ASD and TD) was not significant (*F* (1, 31) = 0.002, *p* = 0.97, partial η^2^ < 0.01). The interaction effect between the group and coordinates condition was significant (*F* (1, 31) = 2.51, *p* = 0.05, partial η^2^ = 0.12). Simple Bonferroni-corrected main effect analysis demonstrated that AR error was significantly larger than ER error in the TD group (*F* (1, 31) = 12.56, *p* = 0.001, partial η^2^ = 0.17), while no significant difference was observed in the ASD group (*F* (1, 31) = 0.37, *p* = 0.55, partial η^2^ < 0.01; Fig 5). There was no significant simple main effect of group on either AR or ER errors (AR errors: *F* (1, 31) = 1.93, *p* = 0.17, partial η^2^ = 0.02, ER errors: *F* (1, 31) = 2.18, *p* = 0.15, partial η^2^ = 0.02).

## Discussion

We examined to what extent individuals with ASD depend on visual information of surrounded environment in feedback-absent reaching behaviour. Results demonstrated that both individuals with ASD and TD were affected by directions of shifted reference frame in ongoing reaching for the target. Although TD individuals were apt to reach in allocentric manner rather than egocentric manner, ASDs did not show this prioritization. Our present findings suggest that preferential utilization of allocentric coordination over egocentric coordination in immediate ongoing movements before acquiring an internal model [4–6] evident in TD individuals but not in individuals with ASD.

The TD participants in the present task likely stored the target location relative to allocentric coordinates to decode the spatial position, whereas the results in ASD participants did not show such prioritization in visually guided reaching behavior. While allocentric spatial representation is typically available from 2 years of age [30], incomplete maturation of allocentric representation has been observed in TD children aged 6–7 years [31]. A previous study suggested impaired utilization of allocentric coordinates relative to decreased use of a third-person perspective to perceive the outside space in individuals with ASD [14]. In relation to such perception, VPT has also been reported to be impaired in ASD, but its appearance depends on the demands of the task [15]. Since allocentric coordinates are useful for precise limb movement [9–13], individuals with TD come to preferentially employ allocentric coordinates compared to egocentric coordinates during development. The development of allocentric coordinates may be immature in individuals with ASD.

Allocentric spatial representation is thought to be processed in the ventral stream of the brain, while the dorsal stream is assumed to process egocentric representation [20]. Recent functional magnetic resonance imaging (fMRI) studies have suggested that the occipito-temporal network is associated with the representation of allocentric coordinates [32–34]. A review of DTI studies found that the ASD brain tended to have fewer white matter tracts that pass through the temporal lobe [21]. Previous studies on the neural basis of motor disabilities in ASD frequently reported functional and/or structural abnormalities in the action observation network [35] involving several frontal regions to the inferior parietal lobule [36, 37]. While the lower connectivity of the brain network specifically in the occipito-temporal network in relation to allocentric coordination has been demonstrated, it is difficult to mention the neural mechanisms of reduced utilization of allocentric coordinates for movement in spatial navigation because neural activation when performing movements in ASD has not yet been elucidated.

Since the participants were asked to memorize the target position for 100 ms before presenting the test RF, one might question whether the results were influenced by individual WM. A meta-analysis of 29 papers on 34 studies of WM reported that WM in ASD individuals was poorer than that of TD individuals [38]. Regarding the participants in the present study, although we confirmed no significant between-group difference in the WM sub-score of the WAIS-III, the effect of shifting the allocentric RF relative to egocentric RF on touch positions was weakened when performing analysis of covariance (ANCOVA) controlling for the influence of individual WM scores in WAIS-III in TDs (*F* (1, 30) = 2.93, *p* = 0.10). On the other hand, the equivalent ANCOVA analysis in ASDs retained the conventional result (see S1 File for more details). WM could be important for visual spatial processing [39, 40] including spatial navigation of limb movements while retaining target positions [41], similar to the tasks in our study. Thus, the individual difference in WM between participants would affect the level of utilization of allocentric RF only in the TD group. Further research examining the influence of individual differences of visuo-spatial WM on utilization in ongoing reaching for memorized targets is necessary to extract the effect of coordination on movements.

Extending our findings demonstrated in an experimentally controlled situation to behavior in naturalistic situations (3D space) should be performed cautiously. In the tasks we employed, participants were required to modulate their own arm movements based on dynamic changes of spatial information in 3-dimensional space. In the test trials, subjects had to perform 3-dimensional spatial navigation towards a visual target that immediately changes its location on a 2D display. Despite the 3-dimensional spatial navigation of arm movement, the target spatial information was simplified; thus, it must vary from that in a natural situation. It is still unknown whether the performance of ASD individuals seen in this experimental situation actually relates to their daily motor skills in natural scenes, such as in handwriting and ball throwing, referring to visual frames. Nishimura et al. [42] tested whether type of background scene could influence reaching movements that require an allocentric RF. The participants performed a reaching task each in the condition where the background picture was upright or inverted. The influence of allocentric coordinates on reaching was apparently reduced when the picture was inverted compared to that with an upright picture. These results suggest that many cues in natural scenes that exist in an ecologically plausible manner would be essential for general activities using allocentric coordination. This is the next step to elucidate how this present finding examined in an unusual situation can be generalized to motor activities performed in natural and/or 3D environments.

We found that individuals with ASD manifest no preferential usage of allocentric as well as egocentric coordinates while individuals with TD apt to be affected by allocentric coordinates in ongoing reaching movement. These characteristics observed in every reach movement may be a basis of difficulty for acquiring effective internal model using visual information for motor control in learning, and contribute to difficulties in visually guided motor function in daily life.

## Supporting information

S1 File (DOCX)
