# Supplementary material for: Decreased utilization of allocentric coordinates during reaching movement in individuals with autism spectrum disorder
Source: PLoS One. 2020 Nov 18;15(11):e0236768. doi: 10.1371/journal.pone.0236768 (PMC7673550; doi:10.1371/journal.pone.0236768)
Supplement: S1 File — (DOCX) [file pone.0236768.s001.docx]

Supporting Information S1

Results of the analysis of covariance (ANCOVA) controlling for the effects of the working memory (WM).

We applied an ANCOVA controlling for the influence of individual WM scores in the WAIS-III to investigate whether WM affected AR and ER error. As a result, we observed a significant main effect of coordinates (*F* (1,30) = 5.03, *p* = 0.03, partial η^2^ = 0.14) and interaction effects between the group and coordinate conditions (*F* (1,30) = 4.50, *p* = 0.04, partial η^2^ = 0.13) as well as the results of a two-way ANOVA, which did not control for WM scores. In addition, we observed no significant simple main effect of the coordinate condition in the ASD group (*F* (1,30) = 2.07, *p* = 0.16, partial η^2^ = 0.03). In terms of the results of TD participants, a simple main effect of coordinate conditions was close to significant (*F* (1,30) = 2.93, *p* = 0.10, partial η^2^ = 0.05), although we observed a significant effect in the two-way ANOVA.
